# Species delimitation in the *Stenocereus griseus* (Cactaceae) species complex reveals a new species, *S*. *huastecorum*

**DOI:** 10.1371/journal.pone.0190385

**Published:** 2018-01-17

**Authors:** Hernán Alvarado-Sizzo, Alejandro Casas, Fabiola Parra, Hilda Julieta Arreola-Nava, Teresa Terrazas, Cristian Sánchez

**Affiliations:** 1 Instituto de Investigaciones en Ecosistemas y Sustentabilidad (IIES), Universidad Nacional Autónoma de México, Morelia, Michoacán, México; 2 Centro De Investigación De Zonas Áridas (CIZA), Universidad Nacional Agraria La Molina, La Molina, Lima, Perú; 3 Centro Universitario de Ciencias Biológicas y Agropecuarias (CUCBA), Universidad de Guadalajara, Zapopan, Jalisco, México; 4 Instituto de Biología (IB), Universidad Nacional Autónoma de México, Coyoacán, Ciudad de México, México; 5 Herbario de la Universidad de La Guajira, Riohacha, La Guajira, Colombia; University of Arkansas, UNITED STATES

## Abstract

The *Stenocereus griseus* species complex (SGSC) has long been considered taxonomically challenging because the number of taxa belonging to the complex and their geographical boundaries remain poorly understood. Bayesian clustering and genetic distance-based methods were used based on nine microsatellite loci in 377 individuals of three main putative species of the complex. The resulting genetic clusters were assessed for ecological niche divergence and areolar morphology, particularly spination patterns. We based our species boundaries on concordance between genetic, ecological, and morphological data, and were able to resolve four species, three of them corresponding to *S*. *pruinosus* from central Mexico, *S*. *laevigatus* from southern Mexico, and *S*. *griseus* from northern South America. A fourth species, previously considered to be *S*. *griseus* and commonly misidentified as *S*. *pruinosus* in northern Mexico showed significant genetic, ecological, and morphological differentiation suggesting that it should be considered a new species, *S*. *huastecorum*, which we describe here. We show that population genetic analyses, ecological niche modeling, and morphological studies are complementary approaches for delimiting species in taxonomically challenging plant groups such as the SGSC.

## Introduction

Morphological characteristics of many cactus species are highly prone to convergent and parallel evolution, as well as losses and reversals [1]. Consequently, there are few synapomorphies for supporting phylogenetic relationships among taxa [2,3]. Molecular data are also limited toimprove the understanding ofevolutionary relations among species because of the limited availability of nuclear markers for this group [4],low plastid sequence divergence [5] due to the relatively recent origin and diversification of the Cactaceae [6], and high rates of homoplasy due to incomplete lineage sorting [7]. Therefore, interspecific and lower taxonomic group divergence times are expected to be relatively short as in the *Pilosocereus aurisetus* complex, a relatively young group of species 0.33–0.87 Myr old [8], and in the genus *Harrisia* which includes species approximately 0.20–0.37 Myr old [9]. Frequently, the taxonomic clusters within these closely related groups of species lack morphological and genetic characters for clearly defining their limits [10].

The *Stenocereus griseus* species complex (SGSC) has long been considered a taxonomically puzzling group of taxa. High species similarity in the complex is evident based on stem and flower morphology, triterpene composition, and other characters [11]. Gibson [12] considered a broad distribution for the complex: *S*. *griseus* from northern Mexico to coastal Venezuela, *S*. *deficiens* in coastal Venezuela, *S*. *pruinosus* in southern Mexico, *S*. *longispinus* in southern Mexico, *S*. *laevigatus* distributed in southernmost Mexico and northern Guatemala, and *S*. *hystrix* in the Greater Antilles. Gibson [12] warned about the extensive overlap of morphological features and geographic distribution of these taxa and pointed out a possible anthropogenic influence on their distribution. In fact, Bravo-Hollis [13] suggested that *S*. *griseus* populations from northern Mexico could have been introduced from Venezuela by humans.

The first comprehensive taxonomic review of the SGSC was accomplished by Arreola-Nava [14] as part of a survey of the whole genus *Stenocereus* in which extensive synonymy was found: *S*. *deficiens* and *S*. *longispinus* were considered synonyms of *S*. *griseus* and *S*. *laevigatus*, respectively, and Antillean *S*. *hystrix* or *S*. *fimbriatus* were considered as illegitimate names for *S*. *peruvianus*. Conversely, *S*. *pruinosus* has remained unchanged. Therefore, four species are currently considered members of the SGSC (Table 1), and it can be included into the larger *S*. *griseus* group that also includes *S*. *fricii* and *S*. *chacalapensis*., Phylogenetic analysis based on the plastid *rpl*16 intron and the whole *trn*L-*trn*F region, along with morphological characters including rib number and stem color [12,13], did not resolve the species in this group [15].

**Table 1:** Synonymies of SGSC according to previous reviews and geographical distributions reported.

Region	Reference		
	Bravo-Hollis [13]	Gibson [12]	Arreola-Nava [15]
Mexico	*S*. *griseus*	*S*. *griseus*	*S*. *griseus*
	*S*. *pruinosus*	*S*. *pruinosus*	*S*. *pruinosus*
	*S*. *laevigatus*	*S*. *longispinus*	*S*. *laevigatus*
		*S*. *laevigatus*	
Greater Antilles		*S*. *hystrix*	*S*. *peruvianus*
South America	*S*. *griseus*	*S*. *griseus*	*S*. *griseus*
		*S*. *deficiens*	

**Fig 1:**
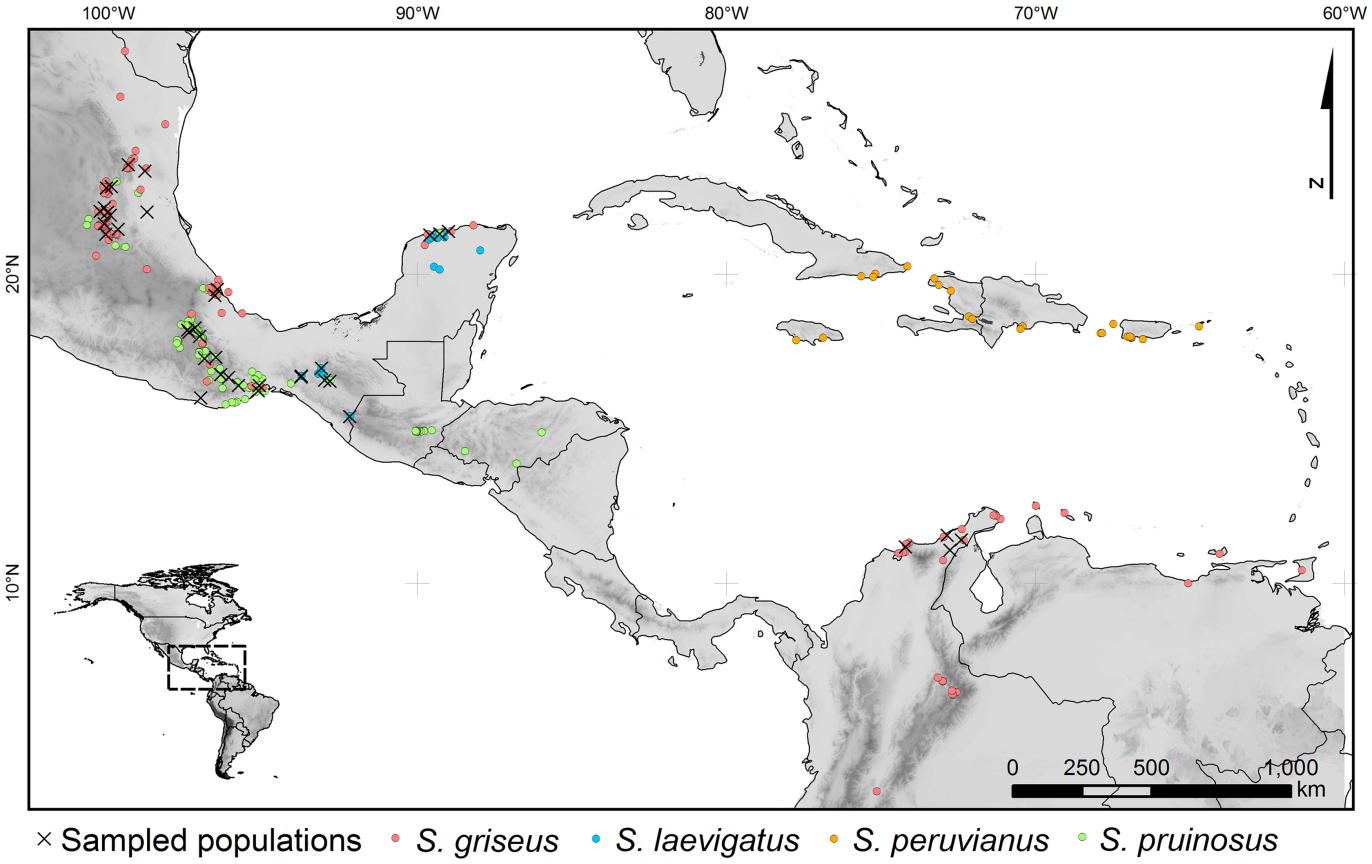
Distribution of the *Stenocereus griseus* species complex (SGSC) taxa, as considered by Arreola-Nava [15].

The lack of agreement may be due to the intricate distributional pattern of the complex: herbarium specimens records describe assorted accessions of *S*. *griseus* and *S*. *pruinosus* in northern and southern Mexico (Chiapas and Yucatán) where a third species, *S*. *laevigatus* (Fig 1) is well represented. Records of co-occurrence can be explained either by misidentification or sympatry.

Other than these taxonomic issues, introgression may be occurring among members of the complex. Parra et al. [16], reported measurable gene flow between *S*. *laevigatus* and *S*. *griseus* in the southern and northern limits of the range of *S*. *pruinosus*. If gene flow is occurring within the SGSC complex, understanding genetic barriers between species will likely shed light on species boundaries throughout their ranges. The distribution of the SGSC includes the Chihuahua and Puebla-Oaxaca diversity centers of cacti [17] interrupted by the Trans-Mexican Volcanic Belt (TMVB), a biogeographic barrier dating back to the Middle Miocene (19.5 to 16 Myr ago)[18]. The Puebla-Oaxaca center includes the Tehuacan Valley region, an early quaternary (2 Myr) sedimentary basin [19] where 64% of Mexican cactus species occur [20] and the Oaxaca Central Valleys. Both regions are isolated by highlands of the Sierra Madre del Sur including the Mixteca Alta and northern and southern ranges in Oaxaca [21], where most SGSC records have originated, mainly those of *S*. *pruinosus* (Fig 1). Tothe east of the Sierra Madre del Sur, the distribution of SGSC continues through the Pacific Coast and the Isthmus of Tehuantepec, a well-known biotic barrier [22] for temperate organisms [23]. For the Cactaceae and specifically the tribe Cacteae, this area is part of a larger Central America [24] or Mexican Pacific Coast [25] region The estimated divergence times for plant taxa across the Isthmus of Tehuantepec ranges from 25 to 4 Myr [26 and references therein], the most recent corresponding to the splitting of *Rhipsalis baccifera* populations [23]. In southern Mexico and Central America, divergence times are associated with the Yucatán Peninsula Karst (3.6 Myr to 18,000 years old) [26], the Central Depression of Chiapas, a 3 Myr old valley formed by the uplift of Chiapas volcanic ranges [27], and the Motagua-Polochic canyons, an active fault at least 15 Myr old [28]. *S*. *peruvianus* is restricted to the Greater Antilles that are 35 Myr old [29]. Finally, a distributional gap is found through the Lesser Antilles, except for the Leeward Islands [30]. The southernmost records of the genus *Stenocereus* are found in Colombia and Venezuela [14], specifically in the Caribbean Coast of North Colombia and Venezuela and the Inter-Andean Valleys [31–33].

The aim of this study is to delimit the SGSC by implementing population genetics clustering methods (Bayesian clustering and distance-based methods) and testing the congruency of these groupings with ecological and morphological analyses. We tested the hypothesis that *S*. *griseus* is a homonym comprising two different taxa, one from Mexico and the other from northern South America. We complemented the species delimitation with spatial references of taxa of the SGSC and its genetic barriers, and the description of the new species *S*. *huastecorum*.

## Materials and methods

### Ethics statement

The permit for collecting plant material in Mexico for studies was provided by national or federal authorities of the Mexican Ministry of Environment and Natural Resources (SEMARNAT) and the National Commission for the Natural Protected Areas (CONANP); in Colombia collection was made under permission of the Ministry of Environment and Sustainable Development (MINAMBIENTE). In addition, we obtained permission from the local authorities and communitarian assemblies of the villages whose territories contained the *Stenocereus* populations we studied. None of the studied taxa are specially protected or endangered species.

### Sampling and study sites

The distribution of the SGSC was compiled from records of biodiversity databases including Tropicos, GBIF, REMIB, and CONABIO, together with information retrieved from herbarium specimens from CHAP, CHAPA, COL, ENCB, IEB, MEXU, UTMC, and XAL (codes following Index Herbariorum [34]). We collected 10–15 cm rib strips from 377 individuals over 35 Mexican populations and four from Colombia (Fig 1); samples were preserved in silica gel for transporting to the laboratory where the tissue was frozen, and then lyophilized in a Christ Alpha 2–4 LD Freeze Dryer (Martin Christ Freeze Dryers, Osterode, Germany).

### Molecular methods

DNA was isolated from either frozen or lyophilized chlorenchyma using the CTAB-based DNA isolation procedure [35]. We tested 15 microsatellite loci previously developed for *Polaskia chichipe* [36,37], *Stenocereus stellatus* [38], and *Stenocereus gummosus* [39]. PCRs with loci suitable for genotyping (see genotyping and markers suitability in the Results section) were carried out in a MultiGene OptiMax (Labnet International, Inc., Edison, NJ, USA) or in a 2700 thermal cycler (Applied Biosystems, Foster City, CA, USA). We pooled three primers from *P*. *chichipe* and one from *S*. *stellatus* in one multiplex reaction, as well as four primers from *S*. *gummosus* in another one. In addition, we separately amplified primer JCS73 isolated from *S*. *stellatus* (see Table A in S1 Appendix for primers and multiplex reactions details). Every reaction was driven to a 5 μL final volume containing 2.5 μL Platinum Multiplex PCR Master Mix (Applied Biosystems, Foster City, CA, USA), 2 μL PCR grade H_2_O, 0.5 μL DNA template (50–200 ng/μL), and a negligible volume of primer mix reaching 70 nM. JCS73 required replacing 0.5 μL of H_2_O by the same G/C enhancer volume in order to assure amplification success. Both multiplex reactions required an annealing temperature of 56°C, while we used 50°C for JCS73; 40 cycles were used in every PCR reaction. Additional cycling conditions were implemented following manufacturer directions.

### Genotyping and marker suitability

Capillary electrophoresis was performed in a 3130*xl* Genetic Analyzer (Applied Biosystems, Foster City, CA, USA). Genotyping was achieved by using the Peakscanner software v1.0 (Applied Biosystems, Foster City, CA, USA), while scoring errors and null alleles were searched through MICRO-CHECKER [40], and linkage disequilibrium tests were performed by using Genepop [41]. Once the suitable marker set was determined, we performed two types of grouping analysis: Bayesian clustering and genetic distance based methods.

### Bayesian clustering

Three Bayesian methods were used: STRUCTURE [42], which performed with one million Markov chain Monte Carlo discarding the first 100 000 as burn-in and testing up to 15 groups,10 iterations each. The group number (*K*) was determined via the Evanno method [43] through the online service STRUCTURE HARVESTER [44]. The Geneland R Package [45] was performed using 100,000 iterations with a 10,000 thinning, testing 20 groups with 10 repetitions each. We considered as group number the highest likely value of the resulting distribution. TESS [46] was run testing from 2 to 20 Kmax, 100 000 sweeps were performed discarding the first 10%; then the top 20% DIC values runs were filtered and averaged by every Kmax in order to use the criterion recommended by François and Durand [47] for choosing the most likely number of groups. Both STRUCTURE and TESS most likely group number (*K* and Kmax, respectively) iterations were analyzed with CLUMPP [48] in order to summarize individual assignment values.

### Distance-based approaches

A Nei’s standard genetic distance [49] matrix was calculated per population with MSA software [50] bootstrapping it 1000 times. Then, the Barrier [51] program was run for testing three barriers (considering four taxa). Finally, a UPGMA phenogram [52] was built in MEGA 6 [53] with the same distance matrix used for Barrier.

### Geostatistics and Bayesian consensus

**Fig 2:**
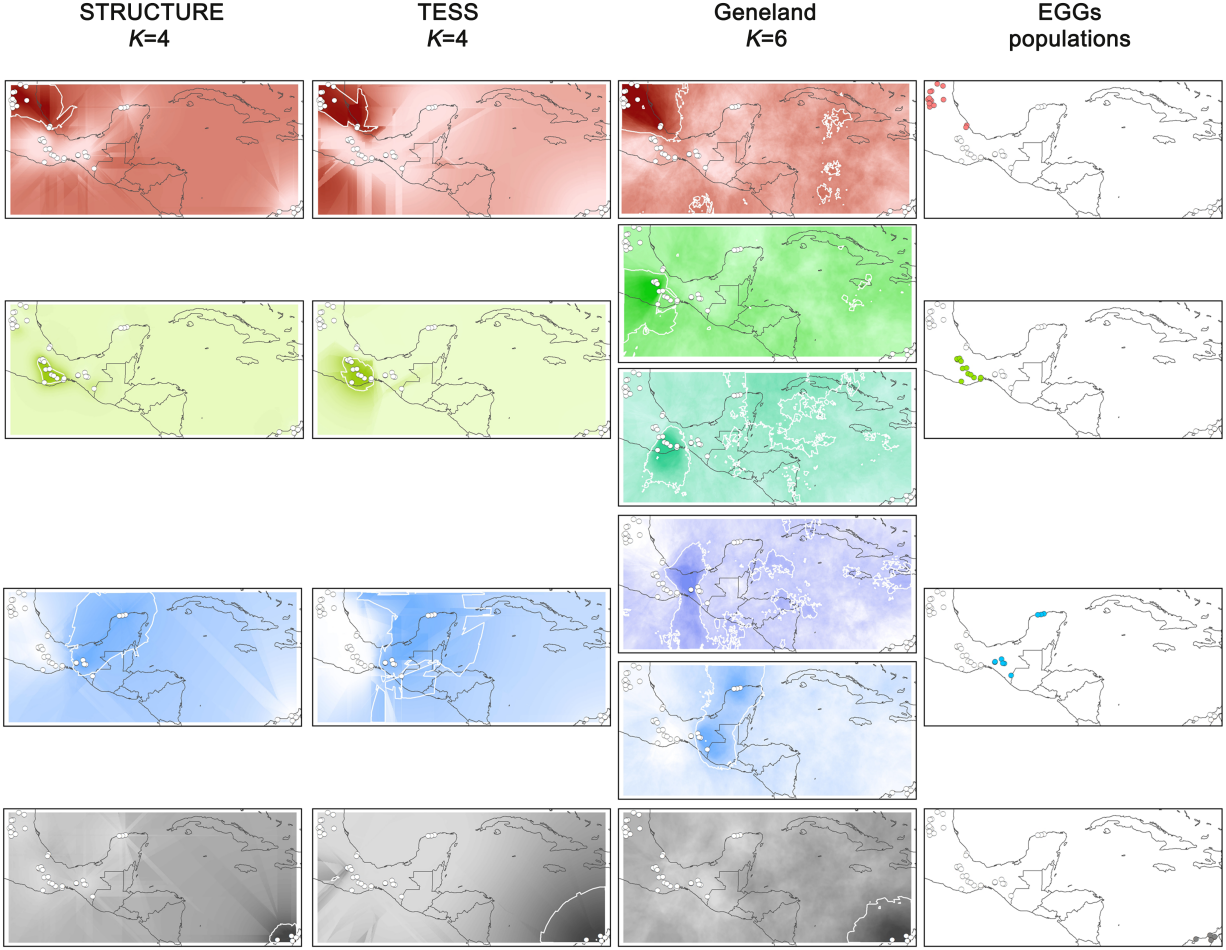
Kriging interpolation of individual Q-matrix by each clustering method. The first three maps columns (left to right) depict the interpolated assignment probability for each method (labeled in the heading) and *K* is the number of groups detected by each. Groups containing the same populations across methods (EGGs) are placed alongside and keep the same color hue, whereas the color gradient saturation represents higher probability: red = *S*. *griseus*-Mexico (here designated *S*. *huastecorum*); green = *S*. *pruinosus* and its subgroups (green shades in Geneland maps); blue = *S*. *laevigatus* and its subgroups (blue shades in Geneland maps), and dark gray = *S*. *griseus*. White bullets represent populations. EGGs populations are represented at the rightmost column by bullets which follow the color code before described.

**Fig 3:**
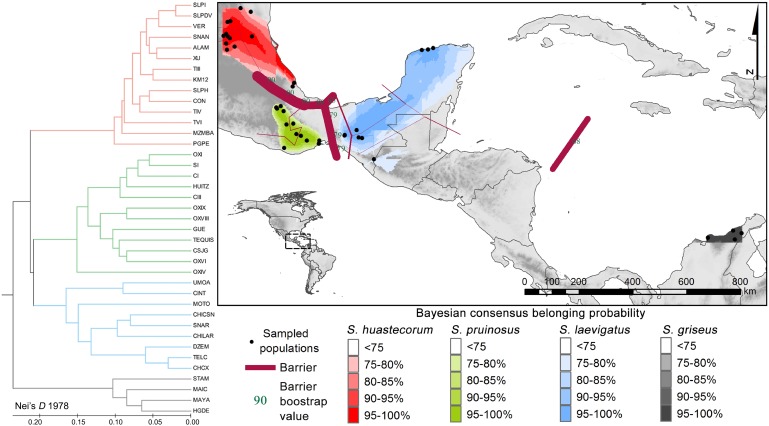
Genetic clustering summary. Color gradients represent BC belonging probability starting from the 75% threshold value, purple bars with green types bootstrap values are the three genetic barriers detected by Barrier, at the left the UPGMA dendrogram following the color code used in the BC.

Kriging interpolation was performed from each Bayesian clustering individual Q-matrix with the ArcMap 10.1 (Redlands, CA, USA) Geostatistical Analyst extension. We defined the genetic groups as high probability areas (≥ 60% of belonging probability) and their equivalence when these comprised the same populations across the three clustering methods (Fig 2). We summed the kriging raster files of equivalent genetic groups (EGGs) in order to obtain a consensus value for each pixel. Finally, each EGG consensus raster was standardized and we projected the top 75% belonging probability as areas (Bayesian consensus, hereafter “BC”), each named after the most common species records included. Distance-based approaches were superimposed over the BC map (Fig 3) in order to spot biogeographic barriers, thus summarizing every genetic method employed.

### Ecological niche modelling (ENM) and comparisons

The SGSC records were obtained by merging the herbarium records with our own collections, and these data were curated by removing points whose values were clearly rounded up or with incorrect geo-references. ENM was performed by Maxent 3.3.3 [54] using 313 records and the 19 bioclimatic variables from Worldclim [55] at 30 arc-second resolution. The variables BIO2 (Mean Diurnal Range), BIO3 (Isothermality), BIO11 (Mean Temperature of Coldest Quarter), BIO13 (Precipitation of Wettest Month), BIO15 (Precipitation Seasonality), and BIO19(Precipitation of Coldest Quarter) that contributed to 95% of the model and showed low correlations were selected to perform individual (species) niche modeling defined by the occurrences intersecting each BC polygon, which contained 59 records for *S*. *griseus*, 111 for *S*. *huastecorum*, 78 for *S*. *laevigatus*, and 65 for *S*. *pruinosus*. For each model, 10,000 random points were used in order to extract bioclimatic data for the environmental background.

In order to evaluate differences among species ecological niches, two methods were used: Contact zone analysis [56] and the multivariate method proposed by McCormack et al. [57]. Comparisons were carried out between spatially contiguous BC polygons (*S*. *pruinosus* vs. *S*. *laevigatus*, and *S*. *laevigatus* vs. *S*. *griseus*), those suspected to be sympatric (*S*. *huastecorum* vs. *S*. *pruinosus*) and homonyms (*S*. *huastecorum* vs. *S*. *griseus*).

### Morphometric analysis

**Fig 4:**
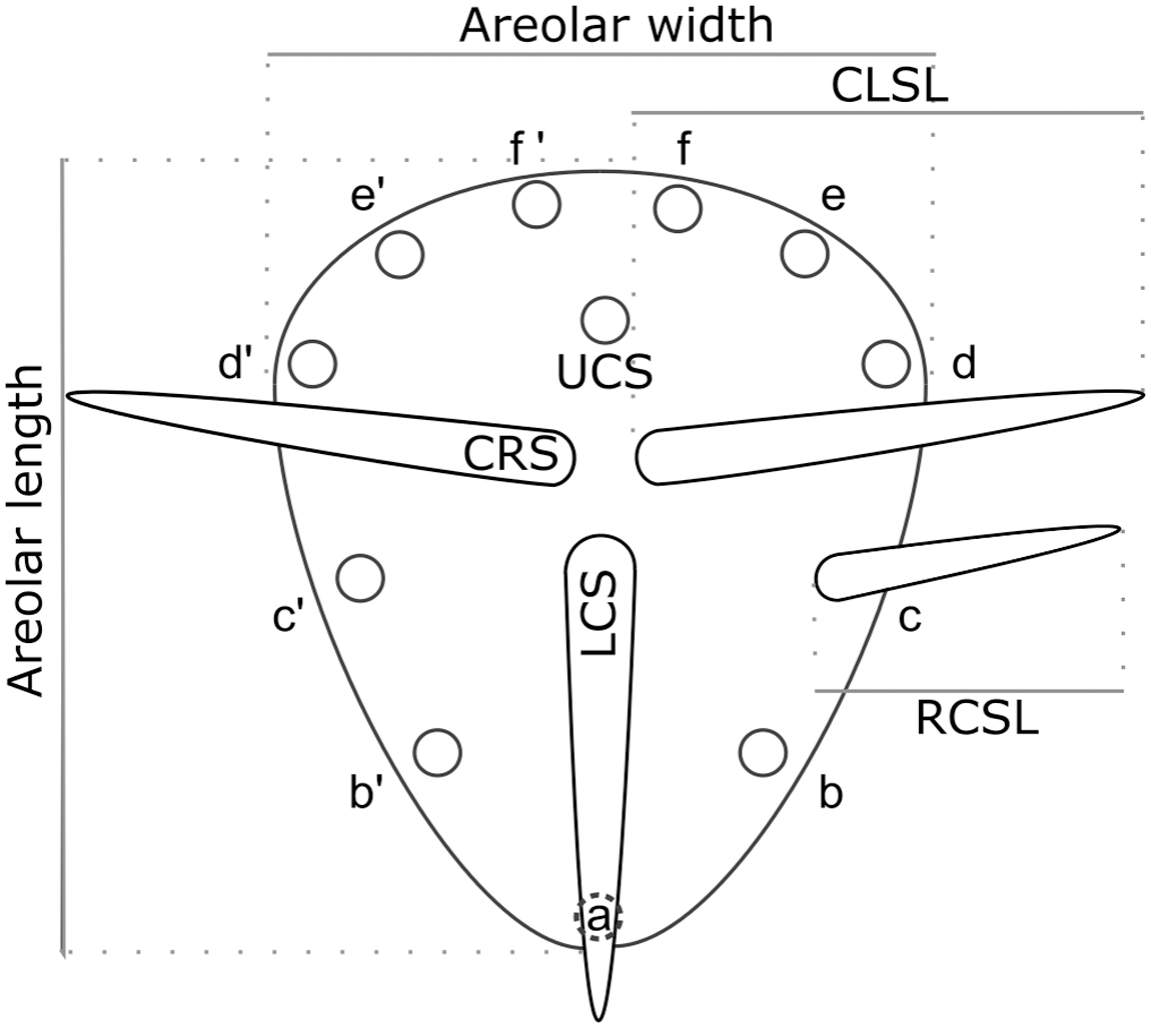
Structural characters measured. CLSL: central-left spine length, CRS: central-right spine, LCS: lower central spine, UCS: upper central spine, RCSL: radial c-homologous spine length, lower-case letters: radial spines (pairs represented by the same letter).

Voucher specimens at MEXU and samples from our collections that contained vegetative portions of stems (subapical areolas may contain additional spines derived from the flower and mature areolas often loss upper peripheral spines) were considered to measure areolar structures, thus reducing the individual numbers to 29 (*S*. *griseus*), 30 (*S*. *huastecorum*), 14 (*S*. *laevigatus*), and 16 (*S*. *pruinosus*). We measured areolar length and width, central spines and a radial homologous spine (named "c-homologous" according to the pattern described by Gibson and Nobel [58]) length, and counted radial and central spines (Fig 4). In order to assess homologous structure comparisons, we followed the areolar development described in [58] as implemented by Tapia et al. [59] in the columnar genus *Neobuxbaumia*. We performed *t*-tests in JMP7 [60] for every variable with clear homology.

### Nomenclature

The electronic version of this article in Portable Document Format (PDF) in a work with an ISSN or ISBN will represent a published work according to the International Code of Nomenclature for algae, fungi, and plants, and hence the new names contained in the electronic publication of a PLOS article are effectively published under that Code from the electronic edition alone, so there is no longer any need to provide printed copies.

In addition, new names contained in this work have been submitted to IPNI, from where they will be made available to the Global Names Index. The IPNI LSIDs can be resolved and the associated information viewed through any standard web browser by appending the LSID contained in this publication to the prefix http://ipni.org/. The online version of this work is archived and available from the digital repositoriesPubMed Central and LOCKSS.

## Results

### Genotyping and marker suitability

Thirteen out of 15 SSRs markers yielded positive PCR products as revealed by 2% agarose gel electrophoresis. However, we discarded four of these loci because they exhibited non-allelic patterns (JCS1 and JCS51), lack of polymorphism (Sgum12), and extensive occurrence of null alleles (Sgum25). After evaluating linkage disequilibrium, we used the final set of loci including Pchi 20, 50, and 54, JCS 49 and 73, as well as Sgum 06, 29, 36, and 39 (for details see S1 Appendix).

### Bayesian clustering

STRUCTURE and TESS detected four EGGs including the same populations (Figures A and B in S2 Appendix), which were designated as *S*. *huastecorum*, *S*. *pruinosus*, *S*. *laevigatus* in northern, central, and southern Mexico, respectively, and *S*. *griseus* from northern Colombia. Geneland, however, showed six groups (Fig C in S2 Appendix), two of them were exactly equivalent to *S*. *huastecorum* and *S*. *griseus*, respectively; whereas two groups (Tehuacán and southern Oaxaca) are contained into *S*. *pruinosus* (Fig 2), finally, *S*. *laevigatus* splits into Cintalapa populations and the remaining populations of this species (Fig 2).

### Distance-based approaches

The Barrier tests revealed three observable geographic barriers: a central/northern barrier supported by high bootstrap values (85–90%), the Isthmus of Tehuantepec (79%), and the third separated the Colombian populations from Mexican taxa, with a 68% support (the purple bars in Fig 3). The UPGMA phenogram (Fig 3) showed four branches matching the STRUCTURE/TESS clustering; the greatest genetic divergence corresponds to the Colombian *S*. *griseus* (Nei’s *D* 0.253–0.293), whereas among the Mexican populations *S*. *huastecorum* differs from *S*. *pruinosus* and *S*. *laevigatus* by 0.262 and 0.204 Nei’s *D*, respectively. These last two species are the closest related taxa, differing from each other by Nei’s *D* = 0.156.

### Ecological niche modeling comparisons

**Fig 5:**
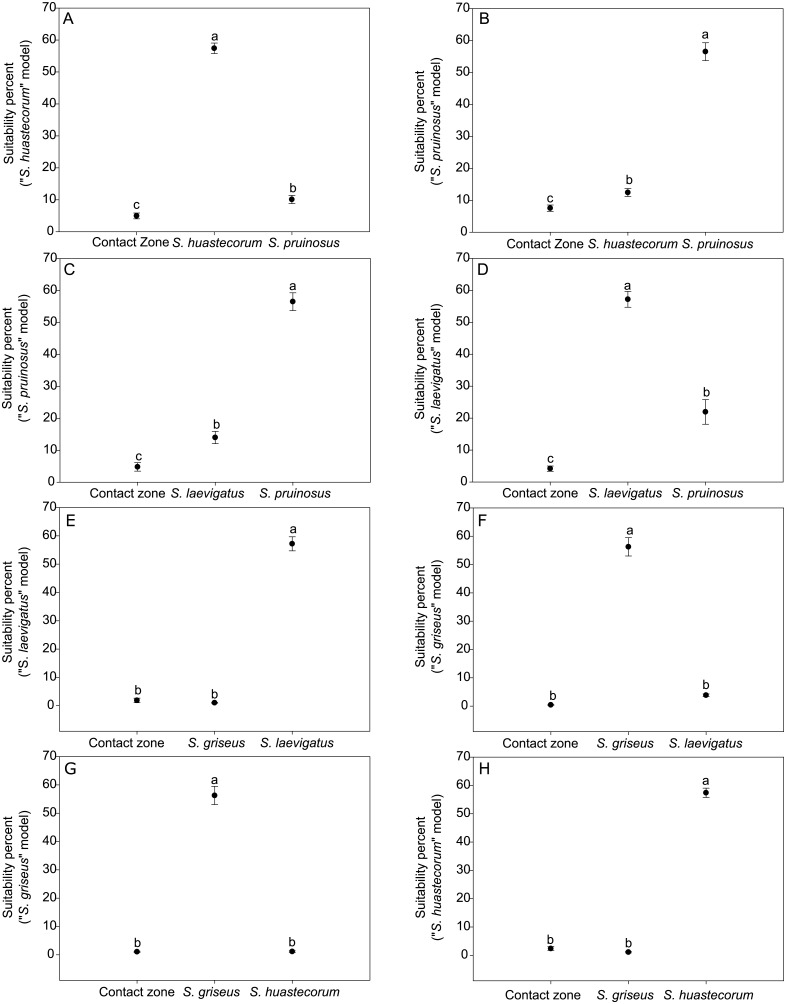
Niche suitability pairwise comparisons based on *t*-tests. Different letters represent levels of significant differences.

The Contact Zone Analysis (CZA) [56], revealed that every pairwise comparison showed clear suitability differences; however, it failed to distinguish the contact zones of *S*. *griseus* with *S*. *laevigatus* and *S*. *huastecorum* (Fig 5E–5H). Conversely, the McCormack et al. [57] method showed general differentiation in principal components 1, 2, and 4, which together explained 74.87% of the variance, and partially for PC 3, which failed in the comparisons by the CZA. PC 5 showed no differentiation between *S*. *huastecorum* vs. *S*. *pruinosus* and *S*. *huastecorum* vs. *S*. *griseus* (Table 2).

**Table 2:** Pairwise comparisons of Ecological niche principal components and two-ways background tests.

Pairwise comparison	Niche axes									
	PC1		PC2		PC3		PC4		PC5	
*S*. *huastecorum* vs *S*. *pruinosus*	**0.3369**		**1.3788**		**0.6422**		**0.5282**		0.1474	
Background tests	0.0419	**1.6743**	**0.7520**	**2.0177**	**1.0944**	**0.3950**	**1.2178**	**0.8736**	**0.2416**	0.0649
*S*. *pruinosus* vs *S*. *laevigatus*	**2.0910**		**0.3313**		**0.3860**		**0.5077**		0.0160	
Background tests	**2.7793**	**1.7122**	**1.0676**	**0.2955**	0.0978	**0.8382**	**0.3178**	**1.1973**	**0.5972**	0.1102
*S*. *laevigatus* vs *S*. *griseus*	**0.6726**		**1.1603**		0.1296		**0.6940**		**0.3032**	
Background tests	0.0157	**1.7122**	**0.3068**	**1.8966**	**0.4104**	**0.6133**	**1.2786**	**0.5033**	**0.2753**	**0.8874**
*S*. *huastecorum* vs *S*. *griseus*	**2.4267**		**2.2078**		0.1266		**0.7144**		**0.4376**	
Background tests	**3.3591**	**1.0893**	**2.8847**	**0.7407**	0.1542	**0.9105**	**1.2990**	**1.0599**	**0.4067**	**0.2252**

Boldfaces represent statistical significance.

### Morphometric analysis

**Fig 6:**
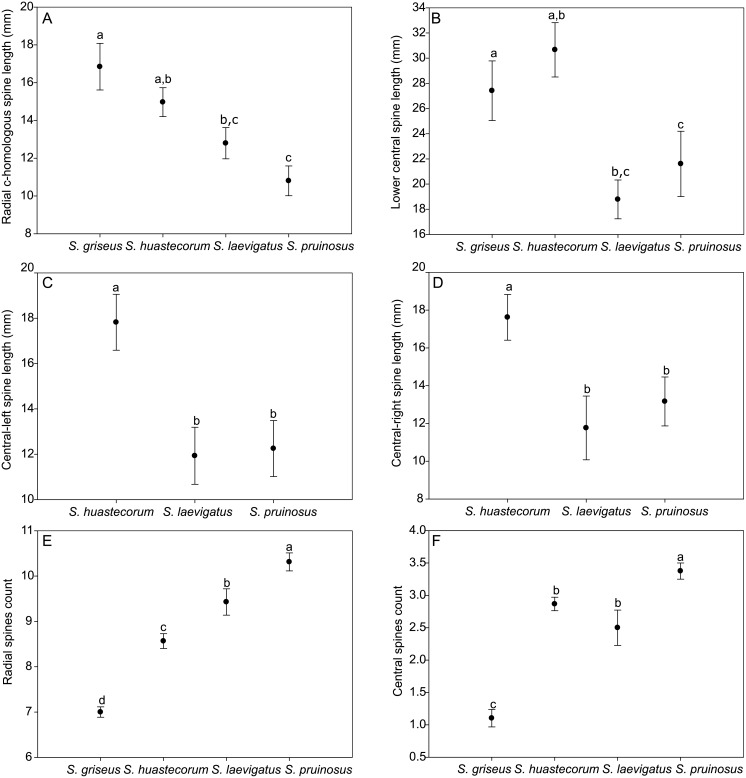
Areolar morphometrics *t*-tests. (A-D) areolar features lengths comparisons, (E-F) spines counts comparisons. Levels not connected by the same letter are significantly different.

Areolar width and length showed no differences between the taxa analyzed. The lower central and radial c-homologous spines length failed to differentiate *S*. *huastecorum* from *S*. *griseus* and *S*. *pruinosus* from *S*. *laevigatus*. Both radial and central spines counting displayed full dissimilarity between species (*t* = 1.989, df = 84, *P*<0.005 and *t* = 1.988, df = 85, *P*<0.05). Since *S*. *griseus* lacks upper left and right central spines, these comparisons could be performed only for the Mexican taxa, and among these only *S*. *huastecorum* differed from both *S*. *pruinosus* and *S*. *laevigatus* (*t* = 2.010, df = 49, *P*<0.05 and *t* = 2.011, df = 48, *P*<0.05) (Fig 6).

### Taxonomy

#### *Stenocereus griseus* (Haw.) Buxb., Botanische Studien 12:100. 1961 [61]. [urn:lsid:ipni.org:names:244634–2:1.3.2.1]

Type: América meridional, (unknown). Neotype (here designated) Colombia, Departamento de La Guajira, Municipio de Uribia, 8 km de Manaure. 31-Enero-1963, C. Saravia T. N°. 2183 (COL98661).

Additional observed specimens: Table A in S3 Appendix.

Candelabraform tree, up to 9 m tall; trunk many times absent, <30 cm tall and <20 cm width; many branches, ascending, about 15 cm width, unconstrained, grayish green to glaucous; first order branching; mucilage cavities not evident in transverse branch sections; ribs 7 to 9, acute in transverse section, straight in longitudinal section, 12–35 mm tall by 11–35 mm wide at the base, without horizontal constriction between areolas within the same rib; areolas 8–28 mm apart each, round to obovate (scutelliform), 6–11.5 mm long and 6–10.6 mm wide, with light-colored trichomes; radial spines 5–7, subulated, divergent, 8–30 mm long, white when young, grayish when mature; 1 central spine, rarely 3, subulated, robust, 9.7–56 mm long, white with reddish base when young, grayish at maturity; subapical flowers, night anthesis remaining opened until the next morning, infundibuliform, 5.6–6 cm long and 4.4–5 cm wide in anthesis; pericarpel cylindrical, green, 11.5–14.5 mm wide, covered with slightly prominent podaria, separated, with wide oblong scales about 1.4–2.3 mm long by 1.5–3.2 mm wide at the base, reddish, areolas presenting light-colored trichomes, spines 7–13 mm long; receptacular tube 30–54 mm long, podaria with decurrent scales, narrow oblong to spatulate, apex obtuse to mucronated, about 4–8 mm wide with few trichomes and spines; outer perianth segments narrow oblong, apex rounded to truncate, 16–20 mm long and 6.5–10 mm wide, green to reddish from the bottom to the top; inner segments oblong to spatulate, entire margin, up to 2.5 cm long and 1 cm wide, white to pinkish-white; stamina included, numerous, arranged in verticillated series; basifix anthers, yellowish, style 23–52 mm long and 9–19 mm wide, white to pinkish white; stigma lobules 8–10, 2–4 mm long, yellowish white; nectar chamber semi-closed by the lower filaments curvature, 11–19 mm long and 5.9–6.7 mm wide, striated; ovary 8–13.3 mm long and 5.6–10.8 mm wide; fruit ovoid, dehiscent when ripe, about 48 mm in polar diameter by 40 mm in equatorial diameter, dark red, covered by areolas with numerous setose spines, about 11–18 mm long, yellowish white, deciduous at maturity, sweet flesh, red; ovoid, black seeds 1.3–2.2 mm long by 0.9–1.2 mm wide.

Common name: In Venezuela “cacto dato”, in Colombia: “cardón”, “cardón guajiro”, "yosú", "panameña", and “iguaraya” (fruit).

Phenology: flowers and fruits are produced over the year, but the peak of production lasts from November to April.

Habitat: *S*. *griseus* grows in tropical deciduous forest and xerophytic scrub alongside with *Cereus* spp., *Prosopis juliflora*, *Bulnesia arborea*, *Ceiba* sp. and *Haematoxylum* sp. From sea level to 1200 meters above sea level.

Discussion: *S*. *griseus* is the sole species of the genus *Stenocereus* to occur in South America. This name was actually a homonym comprising a previously undescribed northern Mexico species (*S*. *huastecorum*) and a second one from northern South America, which shall conserve the name *S*. *griseus* by priority principle.

Distribution: Departments of Boyacá, Huila, La Guajira, Magdalena, and Santander in Colombia; States of Falcón, Lara, Mérida, Sucre, Táchira, Vargas, and Zulia in Venezuela, it is also found in Aruba, Bonaire, and Curaçao islands.

#### *Stenocereus huastecorum* H. Alvarado-Sizzo, H. J. Arreola-Nava, and T. Terrazas. sp. nov. [urn:lsid:ipni.org:names: 77173956–1]

Holotype: México, Estado de Guanajuato, Puerto las Tinajas, terracería entre Puerto de Palmas y Álamos de Martínez. 100°06’10.35” W, 21°28’42.33”N, 15 de Junio de 2016, Hernán Alvarado-Sizzo 350 con I. Torres-García y F. Paz-Guerrero. (MEXU 140542).

Isotype: Hernán Alvarado-Sizzo 350 con I. Torres-García y F. Paz-Guerrero (MEXU 140543).

Additional observed specimens: Table B in S3 Appendix.

**Fig 7:**
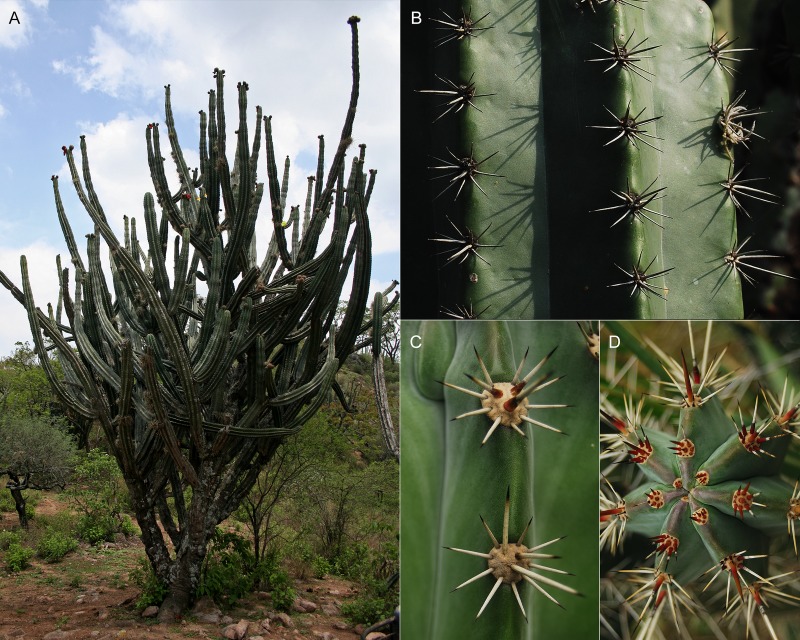
Vegetative features of *S*. *huastecorum*. (A) general aspect of *S*. *huastecorum* (H. Alvarado-Sizzo 350), (B) rib details (H. Alvarado-Sizzo 352), (C) typical young (upper) and mature (lower) areolas (H. Alvarado-Sizzo 245), (D) apex of a young branch. Credits: (A) I. Torres-García, (B-D) H. Alvarado-Sizzo.

**Fig 8:**
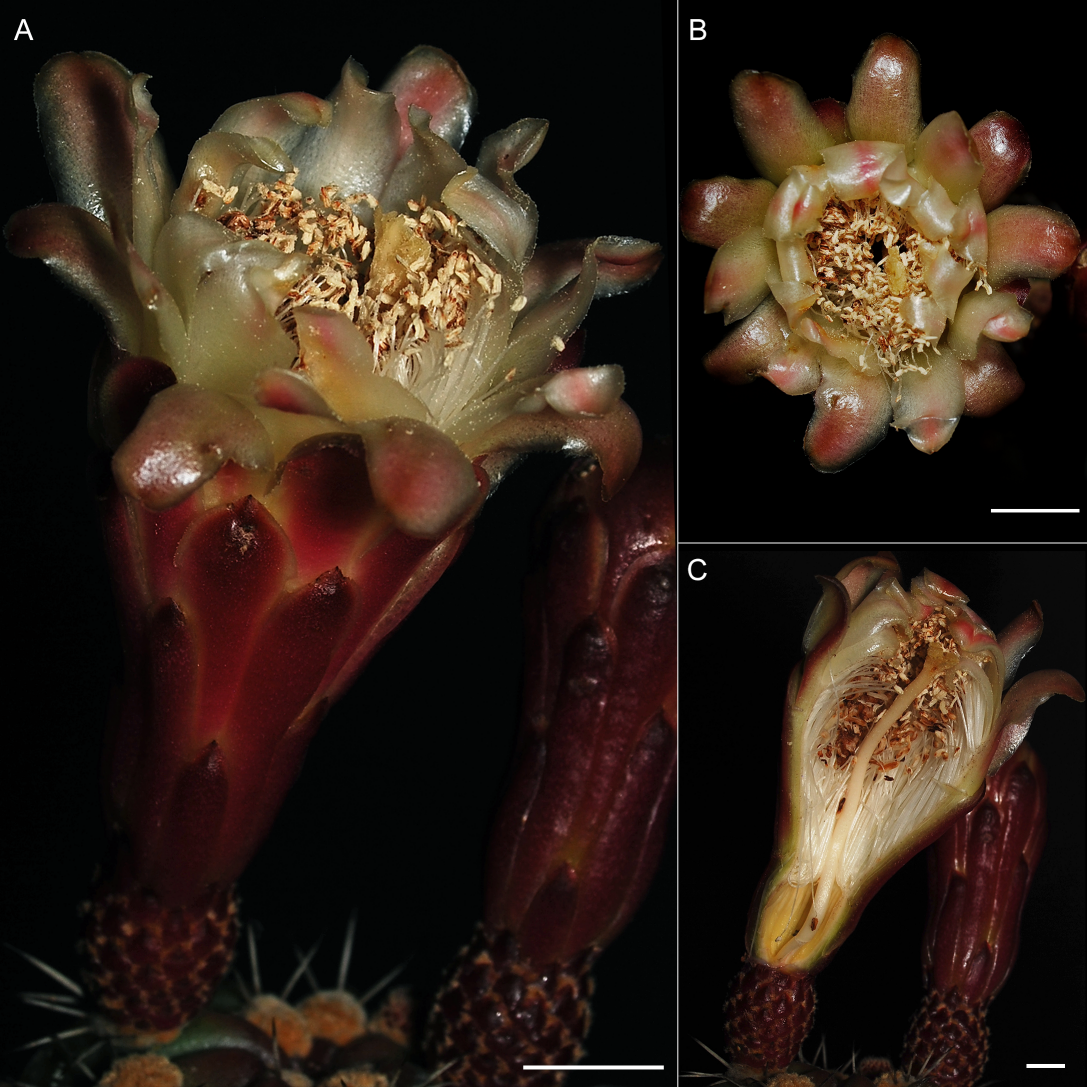
A newly opened *S*. *huastecorum* flower. Lateral (A) and top (B) view, (C) longitudinal cut of the floral tube. (H. Alvarado-Sizzo 350). Credits: H. Alvarado-Sizzo. Scale bars = 1 cm.

**Fig 9:**
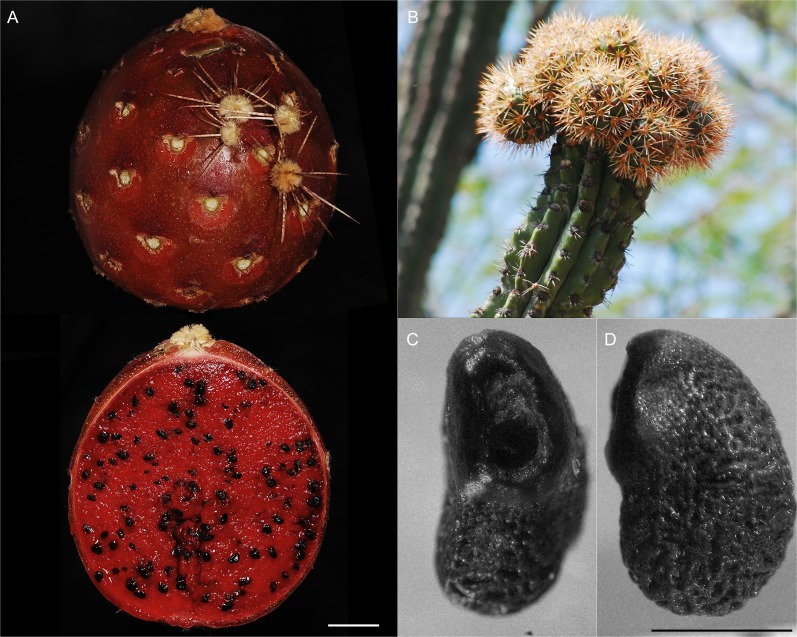
Ripe fruit of *S*. *huastecorum*. Falling areolas (A, upper) and longitudinal cut (A, lower), aspect of a reproductive branch with immature fruits (B), and focus stacking micrograph (4X) of a seed hilum-micropylar region (C) and lateral view(D). Credits: (A, B) H. Alvarado-Sizzo (C) A. González-Murillo & H. Alvarado-Sizzo. Scale bars (A) = 1 cm; (C, D) = 1 mm.

Candelabraform tree, up to 9 m tall; trunk 30–60 cm tall and 20–25 cm width (Fig 7A); many branches, ascending to spreading, up to 6 m long and about 15 cm width, slightly constrained every 20–30 cm, grayish green to glaucous; rarely second order branching; mucilage cavities not evident in transverse branch section; ribs 6 to 8 (Fig 7D), acute in transverse section, slightly sinuated in longitudinal section, 19–35 mm tall by 19–34 mm wide at the base, protrusion between areolas within the same rib (Fig 7B); areolas 11–26 mm apart each, round to obovate (scutelliform), 5.6–11.9 mm long and 8.5–10.2 mm wide, with light-colored trichomes; radial spines 7–9, subulated, divergent, 7.5–23 mm long, white when young, grayish when mature; 3 central spines, rarely 4 (when upper central one is present), subulated, robust, 8.7–36.5 mm long, white with a reddish base when young, grayish at maturity (Fig 7C); subapical flowers, night anthesis remaining opened during the next day, infundibuliform, 5.7–7.3 cm long and 4.2–5.2 cm wide in anthesis (Fig 8A); pericarpel oblate to very wide ovate, deep red, 11.8–15.8 mm wide, covered with slightly prominent podaria, imbricated, with triangular scales about 2.4–3.4 mm long by 2.7–3.9 mm wide at the base, deep red, presenting ocher trichomes; receptacular tube 33–45 mm long, podaria with decurrent scales, narrow oblong to lorate, apex acute to obtuse, about 4–8 mm wide; outer perianth segments narrow oblong to lorate, apex acute to obtuse, 20–30 mm long and 9–11 mm wide, deep red with yellowish imbrication margins; inner segments oblong, entire margin, up to 2.5 cm long and 1 cm wide, yellowish with a central reddish line (Fig 8B); stamina included, numerous, arranged in verticillate series; basifix anthers, yellowish, style 43–50 mm long and 2–2.4 mm wide, yellowish white; stigma lobules 8–10, 4–7 mm long, yellowish white; nectar chamber semi-closed by the lower filaments curvature, 12–15 mm long and 7.6–8 mm wide, striated (Fig 8C); ovary about 6 mm long and 6–7 mm wide; fruit ovoid, up to 10 per reproductive branch apex (Fig 9B), dehiscent when ripe, 57–58 mm in polar diameter by 50–56 mm in equatorial diameter, dark red, covered by areolas with numerous setose spines, about 7–13 mm long, yellowish white with dark tip, deciduous at maturity, sweet flesh, red or orange (Fig 9A); widely ovoid, black seeds 2.5–2.6 mm long by 1.7 mm wide (Fig 9C and 9D).

Common name: "candelabro", "órgano", "pitayo", "pitayo de mayo", and "pitaya" (fruit).

Phenology: flowers and fruits are produced most of the year (except during the winter), but the peak of production lasts from March to July.

Habitat: it inhabits tropical deciduous forest, xerophytic scrubland, thorny scrubland and mezquital, alongside with *Prosopis* spp., *Vachellia* spp., *Larrea* sp., *Myrtillocactus geometrizans*, and *Stenocereus dumortieri*. From 200 to 1600 meters above sea level.

Discussion: *S*. *huastecorum* populations were considered introduced populations of South American *S*. *griseus* by Bravo-Hollis [13]. Moreover, it was also determined as *S*. *pruinosus*; genetic, ecological, and morphological differences demonstrate it deserves its own designation. This species can be distinguished by its spination pattern (7–9 radial spines and 3 central spines), pericarpel color (deep red), and restricted distribution to northern Mexico.

Distribution: endemic to Mexico in the states of Guanajuato, Nuevo León, Querétaro, San Luis Potosí, Tamaulipas, and Veracruz.

Etymology: the name *S*. *huastecorum* follows the previous designation of the genetic entity described by Parra et al. [16] as "huasteca group", given that its distribution coarsely matches the Huasteca ethnolinguistic region, but we rather use the plural genitive ending to emphasize its belonging as a resource for those human groups.

#### *Stenocereus laevigatus* (Salm-Dyck) Buxb., *Botanische Studien* 12:100. 1961 [61]. [urn:lsid:ipni.org:names:244638–2:1.4]

Neotype (designated here): México, Yucatán, Municipio Telchac Puerto, A 2 km S de Telchac Puerto (carretera costera, por carr. A Telchac, al NE de Dzemul. 21°19′ N, 89°16′ W, Selva baja espinosa. Suelo calcáreo. Arbusto ramificado de hasta 3 m de altura; frutos rojizos cuando maduros. A nivel del mar. 26 Jul 1992, H. M. Hernández 2225 con J. S. Flores (MEXU 649000).

Isoneotype (designated here): H. M. Hernández 2225 con J. S. Flores (MEXU 649038).

Additional observed specimens: Table C in S3 Appendix.

Candelabraformtree, 3–8 m tall; defined trunk, 0.5–1 m tall and 15–20 cm wide, dark green with lustrous surface; mucilage cavities not evident in branch transverse sections; ribs 7, rounded in transversal section, straight in longitudinal section, about 3 cm tall by 5 mm wide at the base, without horizontal constriction between areolas within the same rib; areolas 8–23 mm apart, elliptic to obovate, 7.14–13.6 mm long and 5.5–10 mm wide, with light-colored non glandular trichomes; radial spines 7–11, acicular, 5.7–18 (30) mm long, white when young, fading grayish with age; central spines 1–4, deciduous except for the lower one, acicular, larger and more robust than the radial ones, up to 50 mm long, white, turning gray at maturity; lateral or subapical flowers, infundibuliform, 7.8–8 cm long and 4.5–6.5 cm wide when opened, night anthesis; pericarpel globose to ovoid, about 1 cm wide, green, covered with slightly prominent podaria, with short triangular scales, about 2 m long and wide (at the base), greenish with purplish hues, areolas with scarcely dense trichomes, yellowish white, without spines; receptacular tube 2.9–3.2 cm wide, podaria with decurrent scales, oblong, apex acute to mucronate, variable in length, about 0.7 cm wide, with few trichomes; outer perianth segments narrowly oblong to spatulate with acute apex, about 3 cm long and 1 cm wide, green with purplish hues; inner segments oblong to oblanceolate, up to 2.5 cm long and 1 cm wide, entire margin, white to pinkish-white; stamina included, numerous, in verticillate series; yellowish white filaments; basifix anthers, yellowish; style about 6.5 cm long by 2 mm wide; stigma lobules 7, 1.2 cm long, yellowish-white; nectar chamber partially closed by the inner filaments curvature, 2.3 cm long by 0.9 cm wide, striated wall; ovary 1 cm long and 0.8 cm wide; fruit globose to ovoid, dehiscent when ripe, diameter 5 cm, green with reddish hues, covered by areolas with numerous setose spines, about 1.5 cm long, white, deciduous at maturity, sweet flesh, red; ovoid, black seeds, 1.9–2.9 mm long by 1.3–2 mm wide.

Common name: “órgano”, “tuno”.

Phenology: flowers in May, fruits May and June.

Habitat: tropical deciduous forest.

Distribution: states of Chiapas and Yucatán in México, Guatemala.

Discussion: according to Stafleu and Cowan [62], Salm-Dyck’s collections were never herborized. Among Salm-Dyck’s illustrations, however, this name doesn’t appear [63].

#### *Stenocereus pruinosus* (Otto ex Pfeiff.) Buxb., *Botanische Studien* 12:101. 1961 [61]. [urn:lsid:ipni.org:names:244646–2:1.3]

Type. México, cultivated in Berlin Botanical Garden, (unknown).

Neotype (designated here): México, Oaxaca, Municipio de Santiago Huaucuclilla, 10.5 km sobre la terracería Huaucuclilla-Tlalixtlahuaca. 17° 29′ 046 N, 97° 03′ 115 W. Vegetación: Bosque tropical caducifolio. Plantas arborescentes, hasta 4 metros de alto, ramificación basítona, ápice pruinoso. Flores blancas con una franja roja hacia el ápice, tubulares. 10-Marzo-2007, D. A. Aquino García con S. Arias (MEXU 1272791).

Additional observed specimens: Table D in S3 Appendix.

Candelabraform tree, 2–5 m tall; trunk 30–60 cm tall and 15–20 cm width; second and even third order branching, spreading, forming a wide canopy, branches up to 4 m long and 10–15 cm width, grayish green to glaucous; mucilage cavities not evident in branch transverse sections; ribs 6 to 8, acute in transverse section, straight to slightly sinuated in longitudinal section, 20–30 mm tall by 20–30 mm wide at the base; areolas 10–40 mm apart each, round to obovate (scutelliform), 70–80 mm long and wide, with numerous light-colored trichomes; radial spines 7–9, subulated, divergent, 5–30 mm long, white with yellowish base when young, grayish when mature; up to 4 central spines, subulated, robust, up to 40 mm long, white when young, grayish at maturity; subapical or lateral flowers, night anthesis remaining opened until the next day morning, infundibuliform, 8–9.5 cm long and 4.5–6.7 cm wide at anthesis; pericarpel ovoid, green with brownish hues,15–25 mm long and 12–15 mm wide, covered with slightly prominent podaria, imbricated, with triangular scales about 1 mm long and 2 mm wide at the base, greenish, few trichomes, yellowish white; receptacular tube 18–25 mm long, podaria with decurrent scales, oblong, apex obtuse to spatulate, mucronate, about 5–7 mm wide; outer perianth segments narrow obovate, apex round to acute, mucronate, 15–20 mm long and about 13 mm wide, green with brownish margins; inner segments oblong to spatulate, entire margin, 2–4 cm long and 1.5 cm wide, white to pinkish-white; stamina included, numerous, arranged in verticillate series; basifix anthers, yellowish, style 35–45 mm long and 2 mm wide, yellowish white; stigma lobules 8–10, 4–7 mm long, yellowish white; nectar chamber semi-closed by the lower filaments curvature, 10–15 mm long and 5 mm wide, striated walls; ovary about 10 mm long and 5.8 mm wide; fruit ovoid, dehiscent when ripe, 60.2–120 mm in polar diameter by 55–81 mm in equatorial diameter [64], green to purple, covered by areolas with numerous setose spines, about 15 mm long, white, deciduous at maturity, sweet flesh, yellow, orange, red or purple; widely ovoid, black seeds 1.9–2.8 mm long by 1.4–2.1 mm wide.

Common name: "Pitayo", "Pitayo de octubre".

Phenology: flowers during spring (March to May), fruits from April to June with a second reproductive peak between August and October.

Habitat: xerophitic scrubland and tropical deciduous forest, from 300 to 1650 meters above sea level. Grows alongside with *Escontria chiotilla*, *Myrtillocactus geometrizans*, and *Prosopis* sp.

Discussion: Pfeiffer described *Echinocactus pruinosus* from cultivated plants in Berlin Botanical Garden. According to Stafleu and Cowan [62] Pfeiffer’s vouchers were deposited in the KASSEL herbarium, whose collection was destroyed during World War II. *Lemaireocereus longispinus* type, according to Britton & Rose [65] was cultivated in the New York Botanical Garden but now is no longer present.

Distribution: endemic to Mexico, in the states of Guerrero, Oaxaca, and Puebla.

## Discussion

### Homonymy in *Stenocereus griseus*

The populations from northern Mexico and northern South America (red circles in Fig 1), which were previously considered to be *S*. *griseus* consistently are two different entities; these genetic groups (red and gray in Fig 3) are clearly differentiated. Moreover, the South American populations showed high genetic distances (Nei’s *D* ≥0.253≤0.293) compared with the Mexican populations. From an ecological point of view, a net differentiation of the reciprocal niche model suitability was detected (Fig 5G–5H) as well as in the Principal Components 1, 2 and 4 (Table 2). Finally, areolar features show differences in spination patterns, where the populations from northern Mexico have more radial and central spines. Therefore, we consider that there is enough genetic, ecological, and morphological evidence for proposing that populations for the northern Mexico group are a different species. We name this species *S*. *huastecorum* sp. nov., whereas the term *S*. *griseus* should remain for naming the South American populations, according to the priority principle [66]. We found no evidence supporting the Bravo-Hollis [13] hypothesis that the northern Mexico populations belong to the same taxon than the South American *S*. *griseus*. However, it has been recognized that the SGSC are often transported by humans [12,16], and the record of a single event of such type could misguide to such hypothesis. In addition, the incomplete revision in previous works of the South American vouchers and the scarcity of records in Central Colombia and Venezuela certainly favored the acceptance of homonymy.

### *Stenocereus pruinosus* in central Mexico

Genetic delimitation fully supports the statements by Parra et al. [16] about population clusters in northern Mexico (the Huasteca group) and the eastern Tehuantepec Isthmus (the Chiapas group) as species different to *S*. *pruinosus*. Moreover, our study confirmed that the latter has a north-south substructure (green shades clusters in the Geneland column in Fig 2) which corresponds to the Tehuacán-Cuicatlán Valley and the Oaxaca Central Valleys.

*S*. *pruinosus* is separated from *S*. *huastecorum* by a genetic barrier, consistent with the TMVB, and is separated from *S*. *laevigatus* by a second barrier represented by the Isthmus of Tehuantepec, a well-known biogeographic barrier (Fig 3). We did not observe genetic evidence of populations or individuals of *S*. *pruinosus* occurring in northern Mexico. Therefore, we do not consider a sympatric scenario between *S*. *pruinosus* and *S*. *huastecorum* in northern Mexico, but rather a long record of misidentified specimens.

Ecological evidence also provides clear distinction of *S*. *pruinosus* ENMs from those of *S*. *huastecorum* and *S*. *laevigatus* given that their comparisons (Fig 5, Table 2) suggest that these species have different ecological niches. Even though areolar morphology can easily distinguish *S*. *pruinosus* from *S*. *huastecorum* (every variable measured in Fig 6) only spine numbers (Fig 6E and 6F) were able to distinguish *S*. *pruinosus* from *S*. *laevigatus*. Areolar characters, however, may be confusing if developmental features are not taken into account because areolas may lose spines because of flowering events and branch age, or it may be simply deciduous as in central-left and right spines of *S*. *laevigatus*. Poor morphological differentiation is clearly related to the fact that this species pair shows the least interspecific genetic distance (Nei’s *D* = 0.156). This suggests a recent divergence event, which involves the Isthmus of Tehuantepec constraining the distributional range of S. *pruinosus* to the Tehuacán-Cuicatlán and Oaxaca Central Valleys.

### *Stenocereus laevigatus* in southern Mexico

In Chiapas and Yucatán there are up to three SGSC members records (Fig 1). Genetic clustering showed dominance of a single genetic group (Fig 3) that we consider to be *S*. *laevigatus* given that its records are more common than those of other taxa. Secondary genetic structure involving the westernmost populations of Umoa and Cintalapa (Chiapas) was detected by Geneland, Barrier, and UPGMA analyses. This break contrasts with the nesting pattern showed by the Yucatán Peninsula populations within the Chiapas group (Fig 3), even when they are separated by a distributional gap of over 500 km.

We consider the greater genetic divergence within the same region in Chiapas as an indicator of either recent demographic processes or occurrence of artificial selection. Human management is common throughout the distributional range of *S*. *laevigatus*, and it is particularly strong in Chiapas. Management has been previously recognized as a genetic-landscape modifier in *S*. *stellatus* [38], but historic demography and phylogeographic research are still needed to distinguish between biogeographic and human processes.

Even though this species is genetically and ecologically divergent with respect to *S*. *pruinosus*, if only morphology is taken into account, they may arise as cryptic species given that areolar features are very similar or actually identical in young or vegetative branches.

### *Stenocereus huastecorum*, a novel species from northern Mexico

This entity stands as the most cohesive species within the SGSC: its genetic clustering pattern (Fig 3) is apparent regardless the method used. Ecological (Fig 5A, 5B, 5G and 5H; Table 1) and morphological evidence (Fig 6) shows its uniqueness. Therefore, we consider this group deserves the status of a distinct species given that there is enough genetic, ecological, and morphological evidence to distinguish it from *S*. *griseus* (its homonym so far) and *S*. *pruinosus*, a supposed sympatric species.

The distributional range of *S*. *huastecorum* sp. nov. comprises southern Tamaulipas, western San Luis Potosí, northern Querétaro and Guanajuato as well as disjunctive populations in Veracruz on the south slopes of the Trans-Mexican Volcanic Belt, displaying a distributional gap of 350 km. The distribution area is mainly contained in the Sierra Madre Oriental and the Llanura Costera Nororiental (Northeastern Coast Plain) physiographic regions of Mexico, coarsely matching the ethnolinguistic “Huasteca” region after which we name this species.

### Summary of species limits and distribution

Every clustering method agreed in major genetic breaks associated with biogeographic regions. We were able to link the range of *S*. *huastecorum* with the Llanura Costera Nororiental, Sierra Madre Oriental (Huastecan Karst), and the southern Trans-Mexican Volcanic Belt, which seems to be the strongest genetic barrier when considering the whole complex (Fig 3). However, this is arguable for *S*. *huastecorum*, given that its populations dwell on both North and South of the oldest section of the TMVB (19.5 to 16 Myr), that predates the age of the Core Pachycereeae (Pachycereinae+Stenocereinae+*Echinocereus*) at 7 Myr [24]. The distribution of *S*. *pruinosus* is apparently contained in geologically recent lowlands, Tehuacán Valley and Oaxaca’s Central valleys, surrounded by the Sierra Madre Sur, and it reaches the Pacific Coast through the Oaxaca’s Southern Range, a Miocene volcanic sequence which is also older than the age of the tribe [67]. *S*. *laevigatus* is separated from *S*. *pruinosus* by the biotic barrier of the Isthmus of Tehuantepec [22] (Fig 3) which we identified as a genetic barrier, although not common for the Cactaceae; [24,25,68,69]. The core distribution of *S*. laevigatus is associated with the Central Depression of Chiapas and the Yucatecan Karst, specifically with the most recent areas of the Yucatán Península which date to barely 18,000 years [26] suggesting recent colonization. *S*. *griseus* seems to be widespread in Caribbean Coast of North Colombia and Venezuela well as Inter-Andean Valleys, and it is isolated from other SGSC members by the Caribbean Sea and Central America with a singular distributional pattern given that *Stenocereus* and its relatives are clearly North American [24]. Finally to explain both the *S*. *griseus* and Antillean *S*. *peruvianus* distributions, a more complex, biogeographic hypothesis other than human transport [13] needs to be tested. We observed spatial concordance between biogeographic and genetic barriers, well-known barriers (mainly highlands) such as the Trans-Mexican Volcanic Belt or Sierra Madre del Sur are by far older than the genus *Stenocereus*, thus being temporally discordant, whereas extant distribution areas are more recent (less than 4 Myr). This odd pattern may suggest that these ranges are soft barriers, with present-day distributions reflecting historical population refugial dynamics [70], or strong human effects on the genetic landscape considering that *S*. *pruinosus* is a intensively managed resource [71]. Demonstrating that one or a combination of these hypotheses will require a time-calibrated phylogeny not only of the SGSC but of the whole genus *Stenocereus*, as well as a phylogeographic approach within the major lineages.

### Conclusions

We conclude that (1) the SGSC shows clear agreement between genetic and biogeographic regions. These genetic barriers, however, seem to be temporally discordant with geographic barriers, thus making a time-calibrated approach necessary. (2) *Stenocereus griseus* is a homonym of the South American species, which should keep the name by priority, and the new species from northern Mexico is here named *Stenocereus huastecorum*. (3) Co-occurrence of species records represent species misidentification rather than sympatry or admixture. Finally, the use of phylogeographic methods in the SGSC, including populations of Antillean *S*. *peruvianus*, is still needed to find evidence of the historical, anthropogenic, and biogeographic processes that lead to current species distributions.

## Supporting information

S1 Appendix Microsatellites markers and multiplex combinations used. (DOCX)

S2 Appendix Most likely number of groups (K) according to different Bayesian clustering methods. (DOCX)

S3 Appendix Observed specimens of SGSC. (DOCX)
