# Supplementary material for: Species delimitation in the Stenocereus griseus (Cactaceae) species complex reveals a new species, S. huastecorum
Source: PLoS One. 2018 Jan 17;13(1):e0190385. doi: 10.1371/journal.pone.0190385 (PMC5771577; doi:10.1371/journal.pone.0190385)
Supplement: S1 Appendix — (DOCX) [file pone.0190385.s001.docx]

S1 APPENDIX

**Table A. Microsatellites markers and multiplex combinations used**

| Locus | Reference | Original size range (bp) | Obtained size range (bp) | Mix |
| --- | --- | --- | --- | --- |
| Sgum06 | [55] | 74-92 | 78-94 | M1 |
| Sgum29 |  | 80-100 | 86-106 |  |
| Sgum36 |  | 134-156 | 138-150 |  |
| Sgum39 |  | 106-108 | 94-108 |  |
| Pchi20 | [52,53] | 230-256 | 249-277 | M2 |
| Pchi50 |  | 216-230 | 219-237 |  |
| Pchi54 |  | 158-182 | 156-192 |  |
| JCS49 | [54] | 192-230 | 190-232 |  |
| JCS73 |  | 165-219 | 195-215 | S |
