# Supplementary material for: Species delimitation in the Stenocereus griseus (Cactaceae) species complex reveals a new species, S. huastecorum
Source: PLoS One. 2018 Jan 17;13(1):e0190385. doi: 10.1371/journal.pone.0190385 (PMC5771577; doi:10.1371/journal.pone.0190385)
Supplement: S2 Appendix — (DOCX) [file pone.0190385.s002.docx]

S2 APPENDIX

**
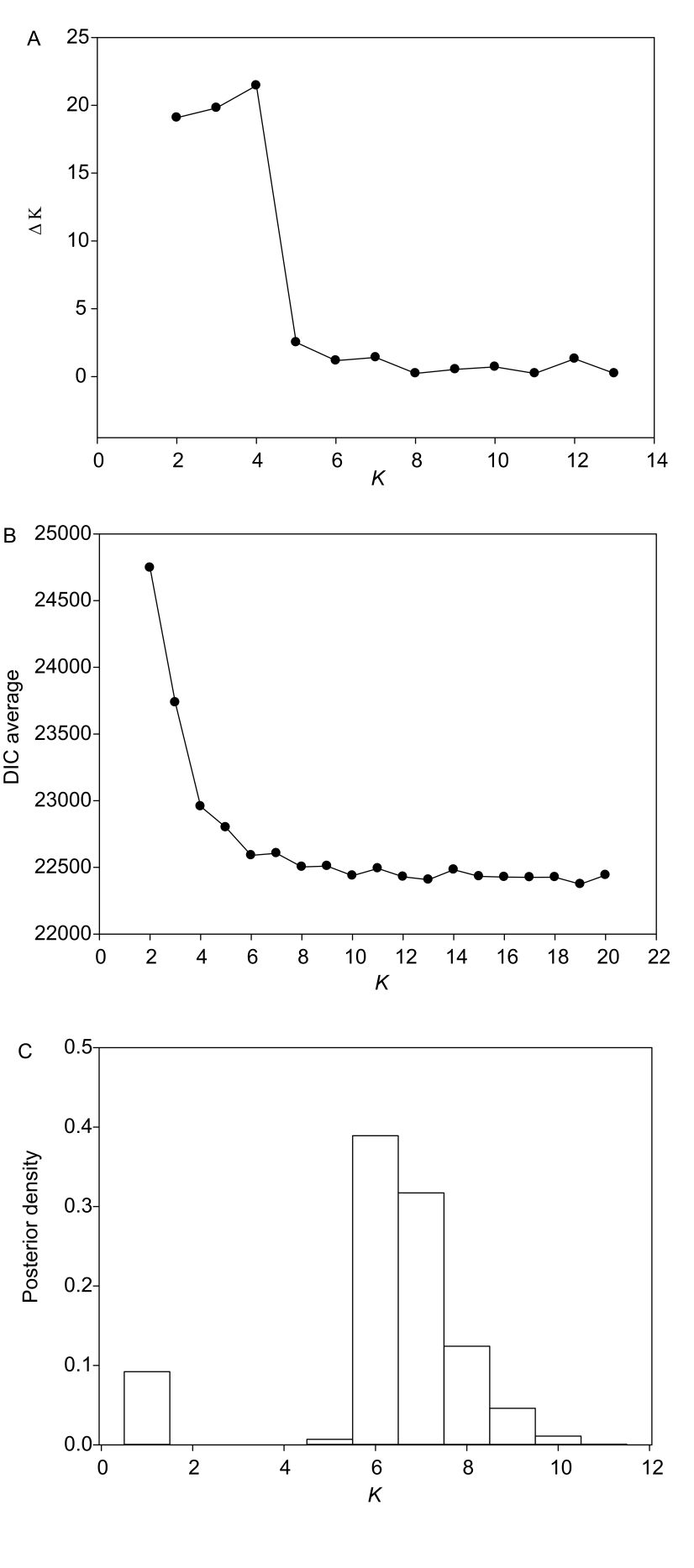
**Most likely number of groups (K) according to different Bayesian clustering methods (A) STRUCTURE, (B) TESS and (C) Geneland
