# Supplementary material for: Species delimitation in the Stenocereus griseus (Cactaceae) species complex reveals a new species, S. huastecorum
Source: PLoS One. 2018 Jan 17;13(1):e0190385. doi: 10.1371/journal.pone.0190385 (PMC5771577; doi:10.1371/journal.pone.0190385)
Supplement: S3 Appendix — (DOCX) [file pone.0190385.s003.docx]

S3 APPENDIX

Observed specimens of SGSC.

**Table A. Observed specimens of *S. griseus* .**

| Herbarium | Voucher | Collector | Collector number | Country |
| --- | --- | --- | --- | --- |
| COL | 103182 | R. Romero-Castañeda | 1937 | Colombia |
| COL | 80314 | R. Romero-Castañeda | 7284 | Colombia |
| COL | 164991 | T. Plowman & W. Davis | 3731 | Colombia |
| COL | 553303 | O. L. Calderón-Rubiano | 163 | Colombia |
| COL | 435153 | A. M. Calcagno & H. Cerda | 87 | Colombia |
| COL | 435143 | A. M. Calcagno | 42 | Colombia |
| COL | 553301 | O. L. Calderón-Rubiano & A. Olaya-Amaya | 106 | Colombia |
| COL | 98645 | C. Saravia T. | 2074 | Colombia |
| COL | 98661 | C. Saravia T. | 2183 | Colombia |
| COL | 47916 | R. Romero-Castañeda | 4371 | Colombia |
| COL | 459000 | A. M. Calcagno & H. Cerda | 48 | Colombia |
| COL | 459007 | A. M. Calcagno | 43 | Colombia |
| COL | 435047 | A. M. Calcagno | 2 | Colombia |
| COL | 463896 | A. M. Calcagno | 45 | Colombia |
| COL | 459002 | A. M. Calcagno & H. Cerda | 44 | Colombia |
| COL | 441423 | J. Hernández R. *et al*. | 2 | Colombia |
| COL | 442581 | J. Hernández R. *et al*. | 2 | Colombia |
| COL | 435066 | A. M. Calcagno | 49 | Colombia |
| COL | 86443 | J. Cuatrecasas & R. Romero-Castañeda | 25448 | Colombia |
| COL | 922662 | C. Saravia & D. Johnson | 594 | Colombia |
| COL | 47951 | R. Romero-Castañeda | 4494 | Colombia |
| COL | 460558 | A. Cadena *et al*. | 2906 | Colombia |
| COL | 435145 | A. M. Calcagno | 42 | Colombia |
| COL | 435055 | A. M. Calcagno & H. Cerda | 87 | Colombia |
| COL | 517668 | S. Albesiano & C. Diaz | 1379 | Colombia |
| COL | 517669 | S. Albesiano & C. Diaz | 1619 | Colombia |
| COL | 463702 | A. M. Calcagno *et al*. | 48 | Colombia |
| COL | 535339 | J. L. Fernández-Alonso *et al*. | 22166 | Colombia |
| COL | 463824 | A. M. Calcagno | 45 | Colombia |
| COL | 435067 | A. M. Calcagno | 49 | Colombia |
| COL | 463701 | A. M. Calcagno *et al*. | 48 | Colombia |
| UTMC | 9566 | A. Barros & J. Jiménez | 27 | Colombia |
| UTMC | 11787 | No collector registered | 3 | Colombia |
| UTMC | 9827 | A. Barros & J. Jiménez | 74 | Colombia |
| UTMC | 9873 | A. Barros & J. Jiménez | 102 | Colombia |
| UTMC | 12949 | A. Barros & J. Jiménez | 6 | Colombia |
| UTMC | 12959 | A. Barros & J. Jiménez | 104 | Colombia |
| UTMC | 12941 | A. Barros & J. Jiménez | 23 | Colombia |
| UTMC | 12942 | A. Barros & J. Jiménez | 18 | Colombia |
| UTMC | 12958 | A. Barros & J. Jiménez | 105 | Colombia |
| UTMC | 12915 | A. Barros & J. Jiménez | 94 | Colombia |
| UTMC | 12916 | A. Barros & J. Jiménez | 25 | Colombia |
| UTMC | 12917 | A. Barros & J. Jiménez | 27 | Colombia |
| UTMC | 12918 | A. Barros & J. Jiménez | 7 | Colombia |
| UTMC | 12930 | A. Barros & J. Jiménez | 32 | Colombia |
| UTMC | 12933 | A. Barros & J. Jiménez | 75 | Colombia |
| UTMC | 12934 | A. Barros & J. Jiménez | 71 | Colombia |

**Table B. Observed specimens of *S. huastecorum.***

| Herbarium | Voucher | Collector | Collector number |
| --- | --- | --- | --- |
| MEXU | 754356 | R.T. Bárcenas & Carlos Gómez H. | 918 |
| MEXU | 697507 | R.T. Bárcenas & Carlos Gómez H. | 694 |
| MEXU | 657754 | R.T. Bárcenas & Carlos Gómez H. | 376 |
| MEXU | 763361 | H.M. Hernández, R.T. Bárcenas & L. Rocha | 3215 |
| MEXU | 830156 | H.M. Hernández, R.T. Bárcenas & L. Rocha | 2797 |
| MEXU | 763366 | H.M. Hernández & Carlos Gómez H. | 3171 |
| MEXU | 769109 | R.T. Bárcenas & Carlos Gómez H. | 1390 |
| MEXU | 858454 | R.T. Bárcenas & Carlos Gómez H. | 1085 |
| MEXU | 1280861 | Carlos Gómez H. & M. E. Correa | 2516 |
| MEXU | 984292 | B. Goettsch | 403 |
| MEXU | 638193 | J. Martínez-Ávalos, J. Jiménez & A. Mora | 415 |
| MEXU | 1227178 | J. A. Barba Montoya | 3 |
| MEXU | 761897 | R.T. Bárcenas & Carlos Gómez H. | 928 |
| MEXU | 697830 | R.T. Bárcenas & Carlos Gómez H. | 688 |
| MEXU | 908160 | R.T. Bárcenas | 47 |
| MEXU | 690585 | R.T. Bárcenas & Carlos Gómez H. | 478 |
| MEXU | 694242 | R.T. Bárcenas & Carlos Gómez H. | 466 |
| MEXU | 756872 | R.T. Bárcenas & Carlos Gómez H. | 742 |
| MEXU | 697515 | R.T. Bárcenas & Carlos Gómez H. | 681 |
| MEXU | 699536 | R.T. Bárcenas & Carlos Gómez H. | 459 |
| MEXU | 647143 | R.T. Bárcenas | 47 |
| MEXU | 756861 | R.T. Bárcenas, H.M. Hernández & Carlos Gómez H. | 842 |
| MEXU | 1233355 | J. A. Barba Montoya | 12 |
| MEXU | 691520 | R.T. Bárcenas & Carlos Gómez H. | 489 |
| MEXU | 697840 | R.T. Bárcenas & Carlos Gómez H. | 698 |
| MEXU | 762187 | H.M. Hernández & Carlos Gómez H. | 823 |
| MEXU | 699622 | R.T. Bárcenas & Carlos Gómez H. | 484 |
| MEXU | 858453 | R.T. Bárcenas & Carlos Gómez H. | 1085 |
| MEXU | 1305349 | Hinton *et al*. | 28291 |
| MEXU | 782674 | R.T. Bárcenas, H.M. Hernández & Carlos Gómez H. | 2997 |
| MEXU | 763362 | R.T. Bárcenas, Carlos Gómez H. & M. Meade | 1543 |
| MEXU | 761851 | R.T. Bárcenas & Carlos Gómez H. | 1384 |
| MEXU | 761973 | R.T. Bárcenas & Carlos Gómez H. | 709 |
| MEXU | 768689 | R.T. Bárcenas, Carlos Gómez H. & M. Meade | 1563 |
| MEXU | 1018130 | B. Goettsch Cabello | 286 |
| MEXU | 191638 | H. Sánchez-Mejorada | 70041 |
| MEXU | 647787 | R.T. Bárcenas & Carlos Gómez H. | 286 |
| MEXU | 670372 | R.T. Bárcenas & Carlos Gómez H. | 355 |
| MEXU | 756974 | R.T. Bárcenas & Carlos Gómez H. | 897 |
| MEXU | 1361642 | H.M. Hernández, Carlos Gómez H. & R.T. Bárcenas | 2797 |
| MEXU | 763687 | Carlos Gómez H. & M. Herrera | 1114 |
| MEXU | 761974 | R.T. Bárcenas & Carlos Gómez H. | 709 |
| MEXU | 761925 | R.T. Bárcenas & Carlos Gómez H. | 1384 |
| MEXU | 782675 | H.M. Hernández, R.T. Bárcenas & Carlos Gómez H. | 2997 |
| MEXU | 763686 | Carlos Gómez H. & M. Herrera | 1114 |
| MEXU | 769115 | R.T. Bárcenas & Carlos Gómez H. | 723 |
| MEXU | 762007 | R.T. Bárcenas & Carlos Gómez H. | 846 |
| MEXU | 769116 | R.T. Bárcenas & Carlos Gómez H. | 723 |
| XAL | 20759 | R. Acosta & I. Acosta | 1581 |
| MEXU | 649080 | R.T. Bárcenas & Carlos Gómez H. | 278 |
| MEXU | 830430 | R.T. Bárcenas | 1276 |
| MEXU | 274431 | B. Hansen *et al*. | 3780 |
| MEXU | 69175 | H. Bravo-Hollis | NN |
| MEXU | 75030 | H. Bravo-Hollis | NN |
| MEXU | 675862 | E. Hernández Xolocotzi | 7674 |
| MEXU | 1145526 | S. Gama-López | 108 |
| MEXU | 1554446 | H. Bravo-Hollis | NN |
| MEXU | 647272 | R. T. Bárcenas | 73 |
| MEXU | 638150 | E. Ventura & E. López | 6651 |
| MEXU | 763356 | Carlos Gómez H. | 1534 |
| MEXU | 1145624 | S. Gama-López | 108 |
| MEXU | 274430 | B. Hansen et al. | 3780 |
| MEXU | 166143 | J. Dorantes et al. | 1096 |
| MEXU | 496659 | L. Scheinvar & J. Dorantes | 851 |
| MEXU | 162416 | A. Lot et al. | 1900 |
| MEXU | 374937 | J. A. Villareal | 1990 |
| MEXU | 346309 | González Elizondo | NN |
| MEXU | 161777 | L. Scheinvar | 857 |
| MEXU | 144044 | H. Sánchez-Mejorada | 70-0301 |
| MEXU | 161769 | C. Delgadillo M. | 42 |
| MEXU | 70509 | H. Bravo-Hollis | NN |
| MEXU | 70508 | H. Bravo-Hollis | NN |
| MEXU | 59644 | H. Bravo-Hollis | NN |
| MEXU | 235403 | P. Cheuva | 199 |
| MEXU | 474787 | H. Sánchez-Mejorada | 3553 |
| MEXU | 159028 | H. Sánchez-Mejorada | 2056 |
| MEXU | 159030 | H. Sánchez-Mejorada | 2086 |
| MEXU | 158995 | H. Sánchez-Mejorada | 2073 |
| MEXU | 159029 | H. Sánchez-Mejorada | 2086 |
| MEXU | 158984 | H. Sánchez-Mejorada | 2084 |
| MEXU | 161779 | C. Delgadillo M. | 28 |
| MEXU | 116842 | C. Delgadillo M. | NN |
| MEXU | 154523 | J. González | 90 |
| MEXU | 161767 | C. Delgadillo M. | 22 |
| MEXU | 161781 | C. Delgadillo M. | 1 |
| MEXU | 235406 | P. Cheuva | 86 |
| MEXU | 118883 | C. Delgadillo M. | NN |
| MEXU | 122121 | C. Delgadillo M. | NN |
| MEXU | 191631 | H. Sánchez-Mejorada | NN |
| MEXU | 118882 | C. Delgadillo M. | NN |
| MEXU | 122122 | C. Delgadillo M. | NN |
| MEXU | 191627 | H. Sánchez-Mejorada | 700402 |
| XAL | NN | R. V. Ortega | 506 |
| XAL | 109055 | M. Nee & Taylor | 29639 |

NN=Not number assigned

**Table C. Observed specimens of *S. laevigatus.***

| Herbarium | Voucher | Collector | Collector number | Country |
| --- | --- | --- | --- | --- |
| MEXU | 649000 | H.M. Hernández & J. S. Flores | 2225 | Mexico |
| MEXU | 649038 | H.M. Hernández & J. S. Flores | 2225 | Mexico |
| MEXU | 1230825 | M. García & F. Ramírez | 473 | Guatemala |
| MEXU | 1148278 | M. Véliz & A. Cobar | 13156 | Guatemala |
| MEXU | 1146860 | M. García & F. Ramírez | 327 | Guatemala |
| MEXU | 1155035 | M. García & F. Ramírez | 286 | Guatemala |
| MEXU | 1146500 | F. Ramírez & M. García | 429 | Guatemala |
| MEXU | 422149 | H. Paniagua | 55 | Guatemala |
| MEXU | 1148561 | M. García & F. Ramírez | 268 | Guatemala |
| MEXU | 444660 | E. Cabrera & H. de Cabrera | 4675 | Mexico |
| MEXU | 238096 | D.E. Breedlove | 42244 | Mexico |
| MEXU | 444659 | E. Cabrera & H. de Cabrera | 4675 | Mexico |
| MEXU | 1168748 | A. Cóbar & F. Ramírez | 357 | Mexico |
| MEXU | 1172749 | F. Ramírez & M. García | 591 | Mexico |
| MEXU | 1270777 | F. Ramírez & M. García | 551 | Mexico |
| MEXU | 1169909 | M. Véliz & M. Pérez | 13302 | Mexico |
| MEXU | 1169910 | M. Véliz & M. Pérez | 13369 | Mexico |
| MEXU | 719963 | J. S. Flores & R. Lira | 10644 | Mexico |
| MEXU | 1148296 | M. García & F. Ramírez | 417 | Guatemala |
| MEXU | 1170025 | A. Cóbar & M. Véliz | 138 | Guatemala |
| MEXU | 1170028 | M. Véliz & M. Pérez | 13396 | Guatemala |
| MEXU | 1254725 | J. J. Linares & C. A. Martínez | 6685 | El Salvador |
| MEXU | 1251328 | J. J. Linares | 6687 | Honduras |
| MEXU | 1170029 | F. Ramírez & M. García | 479 | Guatemala |
| MEXU | 1168660 | A. Cóbar & F. Ramírez | 315 | Guatemala |
| MEXU | 1168842 | A. Cóbar & F. Ramírez | 299 | Guatemala |
| MEXU | 1168431 | M. Véliz & M. Pérez | 13204 | Guatemala |
| MEXU | 1165536 | M. García & F. Ramírez | 488 | Guatemala |
| MEXU | 1251329 | J. J. Linares | 6576 | Honduras |
| MEXU | 1146723 | M. Véliz & F. Ramírez | 12861 | Guatemala |
| MEXU | 1091957 | C. Gómez Hinostrosa | 2238 | Guatemala |
| MEXU | 1182408 | F. Ramírez & A. Cobar | 665 | Guatemala |
| MEXU | 1154859 | M. García & F. Ramírez | 357 | Guatemala |
| MEXU | 1170014 | F. Ramírez & M. García | 488 | Guatemala |
| MEXU | 1270203 | M. Véliz & M. Pérez | 13410 | Guatemala |
| MEXU | 1170079 | M. Véliz & M. Pérez | 13327 | Guatemala |
| MEXU | 1170012 | M. Véliz & M. Pérez | 13338 | Guatemala |
| MEXU | 1252361 | M. García & F. Ramírez | 515 | Guatemala |
| MEXU | 1172738 | F. Ramírez & M. García | 591 | Guatemala |
| MEXU | 1170024 | A. Cóbar & M. Véliz | 142 | Guatemala |
| MEXU | 1170017 | A. Cóbar & M. Véliz | 189 | Guatemala |
| MEXU | 1170016 | F. Ramírez & M. García | 488 | Guatemala |
| MEXU | 1154853 | F. Ramírez et al. | 482 | Guatemala |
| MEXU | 1039163 | M. Véliz et al | 11245 | Guatemala |
| MEXU | 1261846 | A. Cóbar & M. García | 937 | Guatemala |
| MEXU | 1254726 | A. Cóbar & M. García | 850 | Guatemala |
| MEXU | 1148294 | M. Véliz & F. Ramírez | 12916 | Guatemala |
| XAL | 20759 | R. Acosta | 1581 | Guatemala |
| XAL | 28753 | C. Chan et al. | 387 | Mexico |
| XAL | NN | J. I. Calzada et al. | 6533 | Mexico |
| ENCB | NN | M. Laughlin | 839 | Mexico |

NN=Not number assigned

**Table D. Observed specimens of *S. pruinosus.***

| Herbarium | Voucher | Collector number | Collector number |
| --- | --- | --- | --- |
| MEXU | 1326622 | D.A. Aquino | 221 |
| MEXU | 1302116 | S. Arias | 750 |
| MEXU | 1343705 | S.H. Salas | 5894 |
| MEXU | 1326621 | S. Gama López, S. Arias & L. U. Guzmán | 99 |
| MEXU | 1026791 | C. Gómez & A. Cervantes | 2071 |
| MEXU | 1026936 | C. Gómez & A. Cervantes | 2058 |
| MEXU | 931475 | E. Hunn | 568 |
| MEXU | 714527 | R. Torres & L. Cortés | 14369 |
| MEXU | 1276463 | G. Sánchez | 177 |
| MEXU | 1121852 | I. Trejo | 1276 |
| MEXU | 753756 | J. L. Villaseñor, J. I. Calzada & D. Ocaña | 1378 |
| MEXU | 657380 | A. Salinas & E. Martínez Correa | 8172 |
| MEXU | 275261 | B. Leuenberger & C. Schiers | 2524 |
| MEXU | 1347101 | F. Maldonado, A. Sánchez & J. Lucas | 340 |
| MEXU | 837954 | R. García | 285 |
| MEXU | 1233945 | T. Hernández | 43 |
| MEXU | 1269928 | E. Martínez, J. L. Rebolledo y V. Torres | 33454 |
| MEXU | 1274017 | R. Medina | 4694 |
| MEXU | 1121880 | I. Trejo | 1641 |
| MEXU | 1272979 | D.A. Aquino & S. Arias | 57 |
| MEXU | 1272791 | D.A. Aquino & S. Arias | 114 |
| MEXU | 1035553 | C. A. Cruz-Espinosa & E. San Pedro | 535 |
| MEXU | 1035556 | C. A. Cruz-Espinosa, E. Martínez & E. San Pedro | 467 |
| MEXU | 1272797 | D.A. Aquino & S. Arias | 70 |
| MEXU | 59704 | H. Bravo | NN |
| MEXU | 531965 | L.U. Guzmán, S. Gama y S. Arias | 880 |
| MEXU | 10104 | Rose & Painter | NN |
| MEXU | 787532 | A. Salinas-Tovar & R. Martínez | 5929 |
| MEXU | 787547 | A. Salinas-Tovar & R. Martínez | 5929 |
| MEXU | 837955 | A. Salinas-Tovar & R. Martínez | 7121 |
| MEXU | 1249769 | C. A. Cruz-Espinosa | 2147 |
| CHAPA | NN | H. J. Arreola Nava | 1583 |
| CHAPA | NN | Arreola, Terrazas & Arias | 1583 |
| CHAPA | NN | Terrazas & Arias | 449 |
| MEXU | 166778 | H. Sánchez-Mejorada | 724029 |
| MEXU | 98512 | H. Bravo-Hollis | NN |
| MEXU | 549473 | A. Campos | 3549 |
| MEXU | 714528 | A. Campos | 3615 |
| MEXU | 191679 | H. Bravo-Hollis | 62 |
| MEXU | 354725 | R. Cedillo, R. Torres & D. Lorence | 1277 |
| MEXU | 98504 | H. Bravo-Hollis | NN |
| MEXU | 481470 | S. Acosta | 924 |
| MEXU | 98506 | H. Bravo-Hollis | NN |
| MEXU | 485993 | P. Sánchez | 33 |
| MEXU | 528498 | C. Conzatti | 5064 |
| MEXU | 561957 | A. Campos & R. Torres | 4035 |
| MEXU | 75023 | H. Bravo-Hollis | 243 |
| MEXU | 605255 | R. Cedillo | 2236 |
| MEXU | 518144 | S. Gama, U. Guzmán y S. Arias | 34 |
| MEXU | 518127 | L. U. Guzmán et al. | 842 |
| MEXU | 787534 | M. A. Vázquez-Dávila & C. Martínez | 18 |
| MEXU | 177300 | H. Bravo-Hollis | NN |
| MEXU | 59701 | H. Bravo-Hollis | NN |
| MEXU | 518082 | L. U. Guzmán, S. Gama & S. Arias | 781 |
| MEXU | 59703 | H. Bravo-Hollis | NN |
| XAL | NN | A. Saynez Vázquez | 1201 |
| ENCB | NN | A. Saynez Vázquez | 1201 |
| ENCB | NN | D. Franco Estrada | 26 |
| ENCB | NN | J. Rzedowski | 25600 |
| ENCB | NN | P. Sánchez | 33 |
| ENCB | NN | S. Acosta | 924 |

NN=Not number assigned
